# Concealed cysts presenting as 'Club' penis: a case report

**DOI:** 10.1186/s13256-019-2042-7

**Published:** 2019-04-30

**Authors:** T. P. Rajeev, Shalini Krishnan, Arun Menon

**Affiliations:** 1 Department of Urology, K.S. Hegde Charitable Hospital, Nitte (Deemed to be University), Karnataka, 575018 India; 2 Department of Maxillofacial Surgery, A.B. Shetty Memorial Institute of Dental Sciences, Nitte (Deemed to be University), Karnataka, 575018 India

**Keywords:** Epidermoid cysts, Phimosis, Club penis, Inner prepuce, Circumcision

## Abstract

**Background:**

Penile swellings are not very common. They usually present as an obvious lesion visible and palpable either on the penile shaft, glans, or prepuce. Rarely, benign swellings may be concealed by phimosis and can present as “club” penis.

**Case presentation:**

We report the case of a 30-year-old Indian male man who presented with the complaint of difficulty in retracting his foreskin and a club-shaped distal penis. There were palpable lumps on either side of the glans penis which were concealed by the foreskin; hence, a proper preoperative clinical diagnosis was not possible. Circumcision revealed the presence of two discrete cystic swellings from inner prepuce which were excised. Histopathology was suggestive of epidermoid cysts.

**Conclusions:**

Although epidermoid cysts are common cutaneous swellings, they are rarely seen on the penis. They generally present as a small solitary swelling on the penile surface and occurrence at multiple sites is very rare. Epidermoid cysts arising from inner prepuce, hiding within and presenting as club penis have not been reported. Thus, benign lumps should be considered an etiology for phimosis.

## Background

Penile swellings are uncommon disease entities. They range from cutaneous lesions, such as sebaceous cysts and dermoid cysts or malignancies like squamous cell carcinoma, secondary deposits, and sarcoma or rarely a tunical plaque, to urethral diverticulum or a urethral calculus. They clinically appear as a lump that is visible and/or palpable either on the shaft, glans, or prepuce of the penis. When the prepuce becomes non-retractile (phimosis), some penile swellings can be concealed within and not recognizable clinically. If phimosis with a swollen edematous penile tip is present, a hidden malignant tumor of the glans or prepuce may be considered [1].

Here we report a case of a young adult who presented with the complaint of difficulty in retracting his foreskin and a “club”-shaped distal penis. There were palpable lumps on either side of the glans which were concealed by the foreskin; hence, a proper preoperative clinical diagnosis was not possible. At circumcision, two large cystic swellings arising from the inner prepuce were found and excised which were histopathologically proven to be epidermoid cysts. Epidermoid cysts, apart from being a rare penile lesion, have not been reported as a multifocal disease arising from the inner prepuce and disguised as phimosis.

## Case presentation

**Fig. 1:**
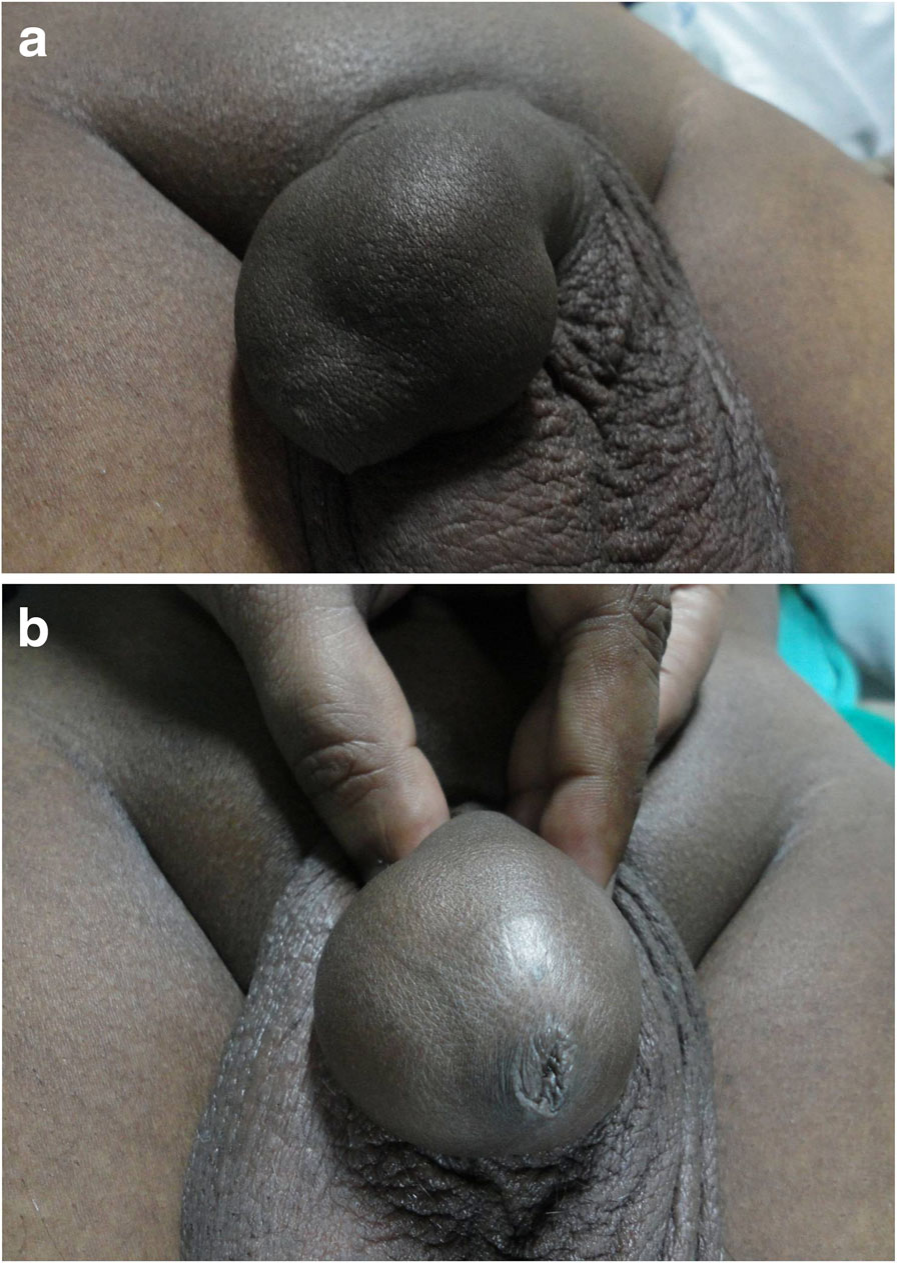
**a** Diffuse bulbous enlargement of distal penis and glans. **b** Unable to retract the prepuce because of hidden swellings underneath

A 30-year-old unmarried Indian male  presented with the complaint of difficulty in retracting his foreskin for the past 3 years. The symptoms were insidious in onset and there was no history of trauma to the glans or foreskin. On genital examination, his distal penis was club shaped with a bulbous and swollen tip. Phimosis was present, but the preputial skin texture appeared normal (Fig. 1a, b). On palpation, a diffuse lump was felt concealed in the foreskin on either side of the glans penis measuring approximately 2–3 cm in size and extending dorsoventrally. It had a smooth outline and was soft in consistency. Further examination of the swelling was not possible because of the phimosis. The differential diagnoses considered at this stage were smegma cyst, preputial cavity fluid collection, and benign cyst.

**Fig. 2:**
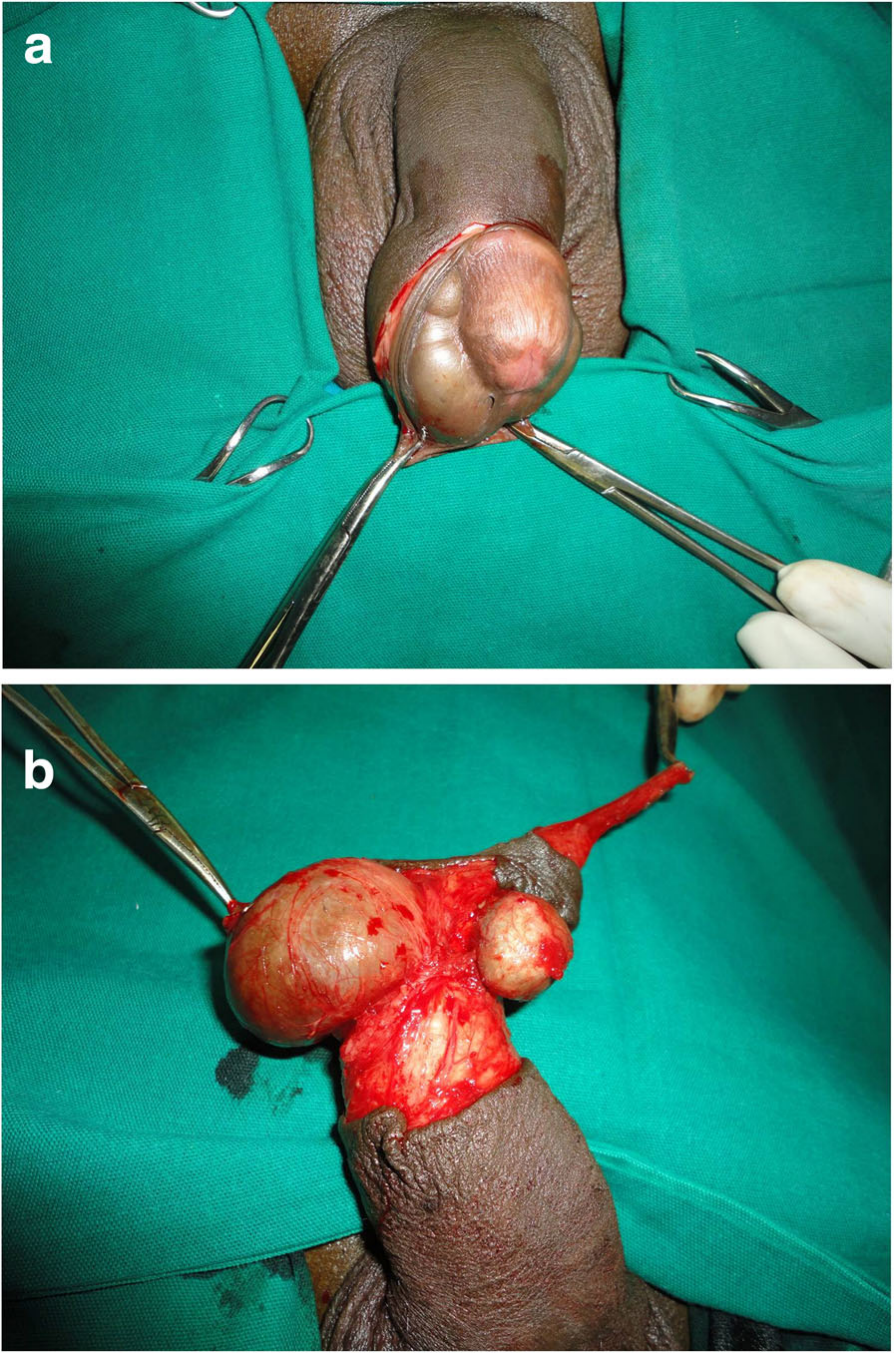
**a** Swellings revealed after incision of foreskin. **b** Multiple cystic swellings visible at ventral surface

**Fig. 3:**
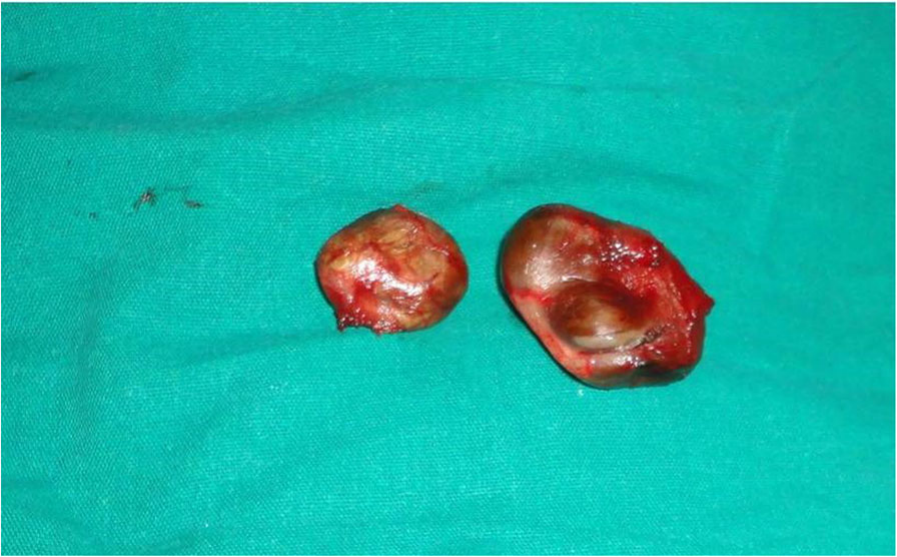
Specimen of the excised swellings

**Fig. 4:**
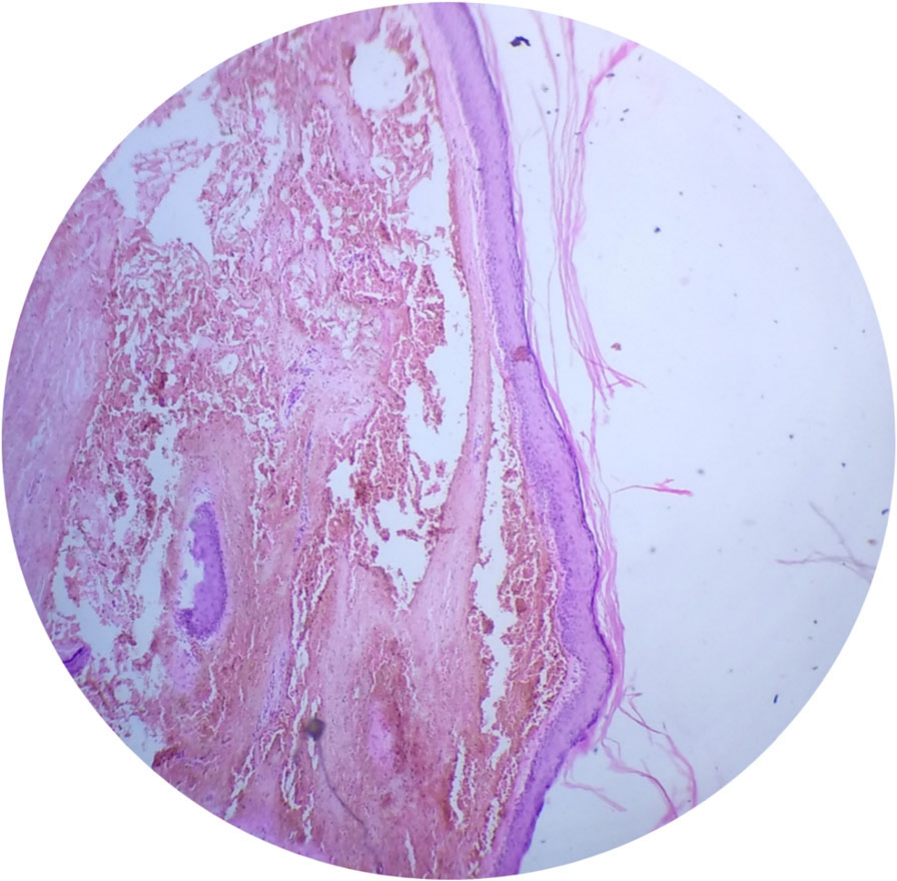
Photomicrograph showing cyst wall lined by stratified squamous epithelium with lamellated keratin

His general medical status was within normal limits and he was taken up for circumcision under spinal anesthesia. The preputial skin was incised and everted which revealed two cystic swellings on the inner preputial surface (Fig. 2a). The right one measured 3.5 × 2.5 × 1.5 cm, irregular in shape, almost extending to the dorsal surface. The left one was 2 × 1.5 × 1 cm, smooth, rounded, and extending to the glans (Fig. 2b). The swellings were excised entirely and circumcision completed (Fig. 3). Our patient had a smooth postoperative recovery and at follow-up the wound had healed primarily. Histopathology revealed both swellings as unilocular cysts with lamellated keratin and lined by stratified squamous epithelium suggestive of epidermoid cysts (Fig. 4).

## Discussion

At birth, the foreskin is fused to the glans and is not retractable until early childhood. The mechanical conditions that prevent foreskin retraction in childhood are a narrow tip of the foreskin or a fusion of its inner surface with the glans penis and a short frenulum [2]. Phimosis is deemed pathological when it causes difficulty in urinating or performing sexual functions. It could also be due to chronic balanoposthitis, untreated diabetes mellitus, lichen sclerosis, repeated catheterization, or obscured carcinoma [3]. Concealed benign swellings that affect the mobility of prepuce, as in this case, are rare.

Epidermoid cysts are the most common benign tumors of the skin that arise from the infundibular portion of a hair follicle spontaneously or subsequent to trauma. They can occur on skin throughout all parts of the body, particularly in the head, face, neck, and thoracoabdominal region [4]. Penile epidermoid cysts are uncommon [4]. Epidermoid cysts of the penis are mostly solitary and multiple ones are very rare. It has been observed that lesions are usually localized on the penile shaft in the dorsal and dorsolateral region [5]. Epidermoid cysts located on the inner prepuce of the penis and concealed at clinical presentation, as in this case, have not been reported.

Epidermoid cysts can be congenital or acquired [5]. Congenital cysts of the penis are rare entities. Development of cysts in the mid-line leads to the speculation that they are mostly congenital and they originate from median raphe. Such cysts are also called median raphe cysts and they occur in close proximity to the frenulum of the penis [6]. Others have suggested that median raphe cysts are a different entity [7]. Trauma or previous surgery can be considered in the etiology of acquired cysts [5]. There was one report of acquired multifocal epidermoid cysts on the penile skin in the literature; the reason for the clinical presentation was cosmetic concerns [5].

Another congenital swelling of the penis is dermoid cyst, which is a true hamartoma and occurs when skin and skin appendages become trapped during fetal development [8]. Dermoid cyst of the glans penis in a toddler, initially thought to be a smegma cyst, was reported [8]. When the foreskin became completely retractile at the age of 4 years, it became apparent that the actual lump was a thick walled cystic lesion in the sub-epithelial tissue of the midline of the glans which shelled out easily at excision. Histopathology revealed epithelial lining and skin appendages. A dermoid cyst of the glans penis is congenital but may present later in life and can mimic other lesions like a solid slow-growing sarcoma [8].

Some syndromes, like Gardner syndrome and basal cell nevus syndrome (Gorlin–Goltz syndrome), are associated with hereditary epidermoid cysts [9]. In Gardner syndrome, epidermoid cysts can occur at multiple sites and at uncommon locations at an earlier age of life with multiple osteomas of facial bones [10].

Penile cysts may be asymptomatic when small. Larger cysts can cause morbidity from inability of preputial retraction, painful intercourse, secondary cyst infection, urethral obstruction, or cosmetic concerns [6]. The differential diagnoses of penile epidermoid cyst include dermoid cysts, median raphe cysts, and urethral diverticula. A voiding cystourethrogram will show communication with urethra in a case of urethral diverticulum. Large and extensive cysts may require ultrasonography or magnetic resonance imaging to depict the anatomical boundaries of the lesion [6].

The treatment of penile epidermoid cyst is complete excision. The lesion may recur after excision if some residual cyst tissue is retained. Our patient required circumcision for the identification of the cyst and its excision.

## Conclusions

Epidermoid cyst of the penis is very rare. It can be multifocal and even originate from inner prepuce. Multiple or large epidermoid cysts at this site can affect the retractability of the foreskin resulting in phimosis. Concealed penile tip swellings are clinically difficult to interpret. Hence, for a young adult with a club-shaped penis and phimosis, benign etiologies such as multiple epidermoid cysts from inner prepuce have to be considered.

## References

[1] Wein AJ, Kavoussi LR, Partin AW, Peters CA. Evaluation of the Urologic patient: history, physical examination, and urinalysis. Campbell-Walsh Urology. 11th ed. Philadelphia: Elsevier; 2015. p. 10.

[2] Oster J. Further fate of the foreskin: Incidence of preputial adhesions, phimosis, and smegma among Danish schoolboys. Arch Dis Child. 1968;43(228):200–3. 10.1136/adc.43.228.200 PMC2019851 5689532

[3] Clark PE, Spiess PE, Agarwal N, Biagioli MC, Eisenberger MA, Greenberg RE, *et al.* Penile Cancer: Clinical Practice Guidelines in Oncology. J Natl Compr Canc Netw. 2013;11(5):594–615. 10.6004/jnccn.2013.0075 PMC4042432 23667209

[4] Singh S, Kaur T. Epidermoid cyst of penis. Indian J Dermatol Venerol Leprol. 2011;77(5):627. 10.4103/0378-6323.84085 21860184

[5] Mehmet K, Coskun S, Serpil O, Ergun U. Multiple epidermoid cysts of penis. Eur J Gen Med. 2012;9(Suppl 1):60–2.

[6] Michihiro S, Masayuki T, Vladimir B, Kota T. Epidermoid cyst of the penis: A case report and review of the literature. Int J Urol. 2000;7(11):431–3. 10.1046/j.1442-2042.2000.00219.x 11144656

[7] Little JS Jr, Keating MA, Rink RC. Median Raphe cysts of the genitalia. J Urol. 1992;148(6):1872–3. 10.1016/s0022-5347(17)37054-4 1433625

[8] Patel RV, Govani D, Qazi A, Haider N. Dermoid cyst of the glans penis in a toddler. BMJ Case Rep. 2014; 10.1136/bcr-2014-204585. PMID-24835812. PMC4025210 24835812

[9] Morice-Picard F, Sévenet N, Bonnet F, Jouary T, Lacombe D, Taieb A. Cutaneous Epidermal Cysts as a Presentation of Gorlin Syndrome. Arch Dermatol. 2009;145(11):1341–3. 10.1001/archdermatol.2009.274 19917979

[10] Mark CL, Scott AB, Andrew MM, Daniel LS. Common benign skin tumours. Am Farm Physician. 2003;67(4):729–38. 12613727

